# Reach further

**DOI:** 10.1530/EC-12-0014

**Published:** 2012-06-08

**Authors:** Jens Sandahl Christiansen

**Affiliations:** 1 Department of EndocrinologyAarhus University HospitalDK 8000, Aarhus CDenmark

Welcome to *Endocrine Connections*, a new Open Access journal offering authors the highest possible visibility for their work and stimulating cross-discipline collaboration. The journal is brought to you by the European Society of Endocrinology (ESE) and the Society for Endocrinology (SfE), working together to further research, education and clinical practice in endocrinology.

The journal will publish original quality research and reviews in all areas of endocrinology, with a focus on papers that have relevance to its related and intersecting disciplines and the wider biomedical community. The endocrine system influences a myriad of cells and tissues within the body. As such endocrine research is often of relevance to other biomedical fields, with examples including, but not limited to, cardiology, dermatology, immunology, neurology and oncology. As an Open Access journal, *Endocrine Connections* allows free permanent access to endocrine research on a global scale, ensuring wide dissemination. Work published in *Endocrine Connections* can be freely accessed by all, providing a unique opportunity to connect with your colleagues in intersecting disciplines. This is something that may not be possible when publishing in traditional endocrinology journals.

As an Open Access journal, papers are published under the Creative Commons Licence 3.0. This means authors retain full copyright of their work and users are free to copy, distribute, transmit and adapt the work for commercial or other purposes, provided the work is properly attributed. It also means authors who are mandated by a funding body to make the paper Open Access can easily comply.

An international editorial board has been appointed, which includes a world-class team of senior editors with a broad range of expertise, who are responsible for handling submissions within their subject area. A broader editorial board is committed to acting as expert reviewers. This editorial structure will allow us to provide rapid responses to authors; we aim to return a decision within 15 days of receipt whilst still maintaining high-quality peer review. Papers will be assessed on the quality and originality of the work presented; a judgement on the perceived level of interest will not be made, as is often traditionally the case. Instead, *Endocrine Connections* will assess papers for broad appeal.

Unedited manuscripts will be published within 24 hours of acceptance and the journal also has a continuous publication model, meaning the final version of record is published as soon as it is ready, without having to wait for assignment to an issue. Together, these factors will ensure that papers will be citable immediately.

We strive to encourage collaboration for the benefit of biomedical science as a whole and, as endocrinologists, we are keenly aware of the need for an interdisciplinary approach to clinical work as well as to clinical and basic research. Please submit your work to the journal and show your support for this new endeavour from ESE and SfE. In return, we will provide fast, high-quality peer review and rapid, instantly citable publication at the same high-quality standards employed by the other official ESE and SfE journals.

We look forward to receiving your submissions.

